# An Investigation of Equine Mesenchymal Stem Cell Characteristics from Different Harvest Sites: More Similar Than Not

**DOI:** 10.3389/fvets.2015.00067

**Published:** 2015-12-07

**Authors:** Karla G. Lombana, Laurie R. Goodrich, Jennifer Nikki Phillips, John David Kisiday, Audrey Ruple-Czerniak, C. Wayne McIlwraith

**Affiliations:** ^1^Gail Holmes Equine Orthopaedic Research Center, College of Veterinary Medicine and Biomedical Science, Colorado State University, Fort Collins, CO, USA; ^2^Department of Comparative Pathobiology, Purdue University, West Lafayette, IN, USA

**Keywords:** stem cells, horse, regeneration, orthopedic, tendon, gene therapy

## Abstract

Diseases of the musculoskeletal system are a major cause of loss of use and retirement in sport horses. The use of bone marrow-derived mesenchymal stem cells (BMDMSCs) for healing of traumatized tissue has gained substantial favor in clinical settings and can assist healing and tissue regeneration in orthopedic injuries. There are two common sites of harvest of BMDMSCs, the sternum and the ilium. Our objective was to determine if any differences exist in BMDMSCs acquired from the sternum and the ilium. We compared the two harvest sites in their propensity to undergo multilineage differentiation, differences in cell surface markers, or gene transduction efficiencies. BMDMSCs were isolated and culture-expanded from 5 ml aspirates of bone marrow from sternum and ilium. The cells were then plated and cultured with appropriate differentiation medium to result in multi-lineage differentiation and cell characteristics were compared between sternal and ilial samples. Cell surface antibody expression of CD11a/18, CD34, CD44, and CD90 were evaluated using flow cytometry, and gene transduction efficiencies were evaluated using GFP scAAV. There were no statistically significant differences in cell characteristics between MSCs cultured from the sternum and the ilium under any circumstances.

## Introduction

Diseases of the musculoskeletal system are a major cause of retirement and euthanasia in horses (1). Currently, a wide range of treatment modalities exist and are used depending on the severity of lameness. Stem cells are the topic of much discussion due to their healing potential in orthopedic injuries (2, 3). Mesenchymal stem cells (MSCs) are thought to increase healing by promoting regeneration of tissues, as MSCs are multi-potent *in vitro* (4–6). Autologous MSCs are commonly acquired from bone marrow aspirates and those bone marrow-derived mesenchymal stem cells (BMDMSCs) have been shown to possess multi-lineage potential *in vitro* (3, 5, 7). A recent study suggests that sources of BMDMSCs affect their propensity to differentiate and migrate in various tissue types (6).

It has become common practice to harvest bone marrow aspirates in the equine when stem cell therapy is pursued. There are two sites of marrow aspiration in the horse: the sternum and wing of the ilium. Currently, the harvest location is completely dependent on clinician preference as there is limited research comparing the cell properties of BMDMSCs from each site. Previous studies have compared growth characteristics, in which BMDMSCs from the sternum and ilium draws had similar growth rates (8). Draw volumes in relationship to differentiation potential has also been investigated, revealing the majority of BMDMSCs are obtained in the first 5 ml of marrow aspirates (9). A recent study indicated cells acquired from the equine sternum were significantly more proliferative than those from the ilium in middle-aged horses (10). Another paper examining overdraw aspirates of bone marrow reported that BMDMSCs from iliac samples proliferate faster than sternum cells (9). A standard has been put forward, which recommends equine MSCs be graded according to a quality standard similar to one in human medicine (11). The standard includes trilineage differentiation potential as well as cell surface marker expression. There have been several studies attempting to determine a specific monoclonal antibody representative of equine MSCs (12, 13), and to date, there are several cell surface markers thought to indicate multipotency of MSCs.

Mesenchymal stem cells are considered a treatment of choice for tendon defects (2, 14–16) as they allow for appropriate differentiation of fiber matrices and appear to reduce the incidence of re-injury. Evaluation of tenogenesis *in vitro* has proven challenging (17, 18) but has recently been successful (6, 19) with the addition of bone morphogenic protein 12 (BMP-12) in monolayer. It was therefore an objective of this study to evaluate tenogenic capacity *in vitro* in addition to classic tri-lineage evaluation.

The therapeutic potential of MSCs is also known to be enhanced through genetic modification (20, 21). Gene therapy offers a unique opportunity to influence the growth factors surrounding orthopedic defects, and is of much focus in current research. This study also examined genetic transduction potential of BMDMSCs acquired from sternum and ilium, to determine the differences in efficiencies critical to future endeavors. Therefore, the objective of this study was to compare the trilineage potential of BMDMSCs harvested from sternum and ilium and further to examine tenogenesis, cell surface markers, and gene therapeutic potential. Our hypothesis was that there would be minimal differences between aspiration locations when the cell characteristics of 5 ml samples were compared in young (2–5 years old) horses.

## Materials and Methods

Bone marrow aspirates from sternum and ilium were acquired from nine horses aged between 2 and 5 years. Red blood cells were removed with centrifugation and cultured overnight in supplemented DMEM (Invitrogen, Grand Island, NY, USA) [10% FBS (Fisher, El Paso, TX, USA), 10,000 U/ml Pencillin–Streptomyocin–Amphotoricn B (Invitrogen, Grand Island, NY, USA) (PSA), 1N HEPES (Invitrogen, Grand Island, NY, USA)]. Media was changed after 24 h, and colonies were observed after 7–10 days in culture. Once colonies were established, cells were cultured in low glucose αMEM (Invitrogen, Grand Island, NY, USA) supplemented with 10%FBS, 10,000 U/ml PSA, 1N HEPES, and 2 ng/ml FGF (R&D Systems, Minneapolis, MN, USA). Cells were passaged three times in monolayer before being cryogenically preserved for evaluation. Passage three cells were recovered overnight and plated to a confluency of 50,000 cells/cm^2^ for differentiation assessments unless otherwise indicated.

### Adipogenesis

Cells from donors (*n* = 9) were treated with DMEM containing 10% fetal bovine serum (FBS), 10,000 U/ml PSA, 1N HEPES, 0.5 mM isobutyl-methylxanthine (Sigma-Aldrich, St. Louis, MO, USA), 1 μM dexamethasone (Sigma-Aldrich, St. Louis, MO, USA), 10 μM insulin (Sigma-Aldrich, St. Louis, MO, USA), and 200 μM indomethacin (Sigma-Aldrich, St. Louis, MO, USA). Negative controls were grown in DMEM with 10% FBS, 10,000 U/ml PSA, and 1N HEPES. Cells were maintained for 14 days and then stained with Oil Red O (Sigma-Aldrich, St. Louis, MO, USA) to detect lipid deposits.

### Osteogenesis

Cells from donors (*n* = 9) were treated with DMEM containing 10% FBS, 10,000 U/ml PSA, 1N HEPES, 0.1 μM dexamethasone, 5 mM beta-glycerol phosphate (Sigma-Aldrich, St. Louis, MO, USA), and 170 μM ascorbic acid (Sigma-Aldrich, St. Louis, MO, USA). Negative controls were grown in DMEM containing 10% FBS, 10,000 U/ml PSA, and 1N HEPES. Cells were maintained for 14 days and then stained with alizarin red and alkaline phosphatase to detect mineralization changes consistent with bone development. Alkaline phosphatase production was quantified from cell lysates with an ELISA kit (Anaspec). Cell lysates were also evaluated for gene expression of alkaline phosphatase (Alk Phos), collagen type III (Col 3), osteocalcin, osteonectin, and runt-related transcription factor 2 (RUNX2) as described below.

### Chondrogenesis

Cells from donors (*n* = 9) were suspended in a 2% low melting agarose gel (Invitrogen, Grand Island, NY, USA) at a concentration of 10 million cells/ml. Cell wells were cultured in supplemented DMEM containing 1% ITS+ (Sigma-Aldrich, St. Louis, MO, USA), non-essential amino acids (Sigma-Aldrich, St. Louis, MO, USA), 100 nM dexamethasone, 10 µM ascorbate-2-phosphate (Sigma-Aldrich, St. Louis, MO, USA), and 5 ng/ml TGFβ (R&D Systems, Minneapolis, MN, USA) for 14 days. Toluidine blue was used to detect extracellular matrix in gel samples, which were also papain digested for total glycosaminoglycan (GAG) using a DMMB assay.

### Tenogenesis

Cells from donors (*n* = 9) were cultured in supplemented DMEM containing 50 ng/ml of recombinant BMP12 (Sigma-Aldrich, St. Louis, MO, USA) (rBMP12), 10,000 U/ml PSA, and 1N HEPES for 14 and 21 days. Negative samples were cultured in media containing all supplements listed above except rBMP12. Samples were evaluated for gene expression of collagen type I (COL I), scleraxis, and tenascin as described below.

### Cell Surface Markers

Cells from donors (*n* = 9) were plated to a confluency of 35,000 cells/cm^2^ and cultured to 80% confluency in DMEM with 10% FBS, 10,000 U/ml PSA, and 1N HEPES. Accumax (Sigma-Aldrich, St. Louis, MO, USA) was used to remove cells from monolayer, and counts were determined using a hemacytometer. Cells were incubated for 45 min at room temperature with primary antibodies for CD11a/18 (AbD Serotec, Raleigh, NC, USA), CD34 (Neuromics, Edina, MN, USA), CD44 (AbD Serotec, Raleigh, NC, USA), and CD90 (Veterinary Medical Research and Development, Pullman, WA, USA) at a concentration of 2.5 × 10^5^ cells/ml. Samples were rinsed with PBS and placed in a secondary IgG, FITC antibody for 45 min at room temperature. Propidium iodide was used to determine the gating region of cells undergoing active growth (i.e., S2/G2/M) as described by Radcliffe et al. (12). Flow cytometry was used to determine the amount of fluorescence for each iliac and sternal MSC sample. CD73 and CD105 marker expressions were determined with rtPCR (data not shown).

### rtPCR

RNA was extracted from samples using an RNeasy kit (Qiagen, Valencia, CA, USA). RNA was reverse transcribed into cDNA as per manufacturer’s instructions (Invitrogen, Grand Island, NY, USA). Tenogenic samples were evaluated at D14 and D21 for gene expression of Col I^I^, scleraxis^II^, and tenascin^III^. Osteogenic samples were evaluated for gene expression of Alkaline Phosphatase^IV^ (Alk Phos), collagen type3^V^ (Col III), osteocalcin^VI^, osteonectin^VII^ and runt-related transcription factor 2^VIII^ (RUNX2). All samples were normalized to an 18S housekeeping gene and were expressed relative to the non-treated control for each sample site.

**Table:** 

Primer sequences		
I	Col1	Forward – ATTTCCGTGCCTGGCCCCATG, Reverse – GCCTTGGAAACCTTGGGGAC
II	Scleraxis	Forward – CTGAGCTGACCCCAGCACTT, Reverse – CCAGAAGAAAACCCAGGTAGGA
III	Tenscin	Forward – CCGGAATATGAATAAAGAAGACGAA, Reverse – CGTACTCTTGCCCAGGAGCTA
IV	ALP	Forward – AAGCACTCTCACTACATCTGGAACCGG, Reverse – GCTCAAAGAGACCCAAGAGGTAATCC
V	Col 3	Forward – GGTCAGTCCTATGCGGATAGAGA, Reverse – CAGAGAACAGATCCTGAGTCACAGA
VI	OCN	Forward – AGAGGTGCAGCCTTCGTGTCCA, Reverse – GCTCCCAGCCAATGATCCAGGTA
VII	OSTN	Forward – CCCCCGGCAATTTCATG, Reverse – AAGCGGTTCCAGTGCTTGAT
VII	RUNX2	Forward – GGCGCATTTCAGATGATGACACTG, Reverse – AGCGGCTCTCAGTGAGGGATGA

### Gene Transduction

Transduction efficiencies were determined for iliac and sternal cells using a scAAV, serotype 2, expressing GFP (green fluorescent protein). Cells were plated at 60,000 cells/cm^2^ in supplemented DMEM and cultured overnight. GFP–scAAVV2 was used to transduce cells at a concentration of 8,000 viral particles/cells (vp/cell) for 3 h in non-supplemented DMEM. Fresh DMEM with 10% FBS, 10,000 U/ml PSA, and 1N HEPES was then added to the cells, and they were cultured for 4 days. Cells were removed from culture using Accumax and evaluated with flow cytometry.

### Statistical Analysis

Paired *t*-tests were performed on all quantifiable assays. Significance was determined when *p* was ≤ 0.05.

This study was carried out in accordance with the recommendations of The Institutional Animal Care and Use Committee. The protocol was approved by the Colorado State University Institutional Animal Care and Use Committee.

## Results

**Figure 1:**
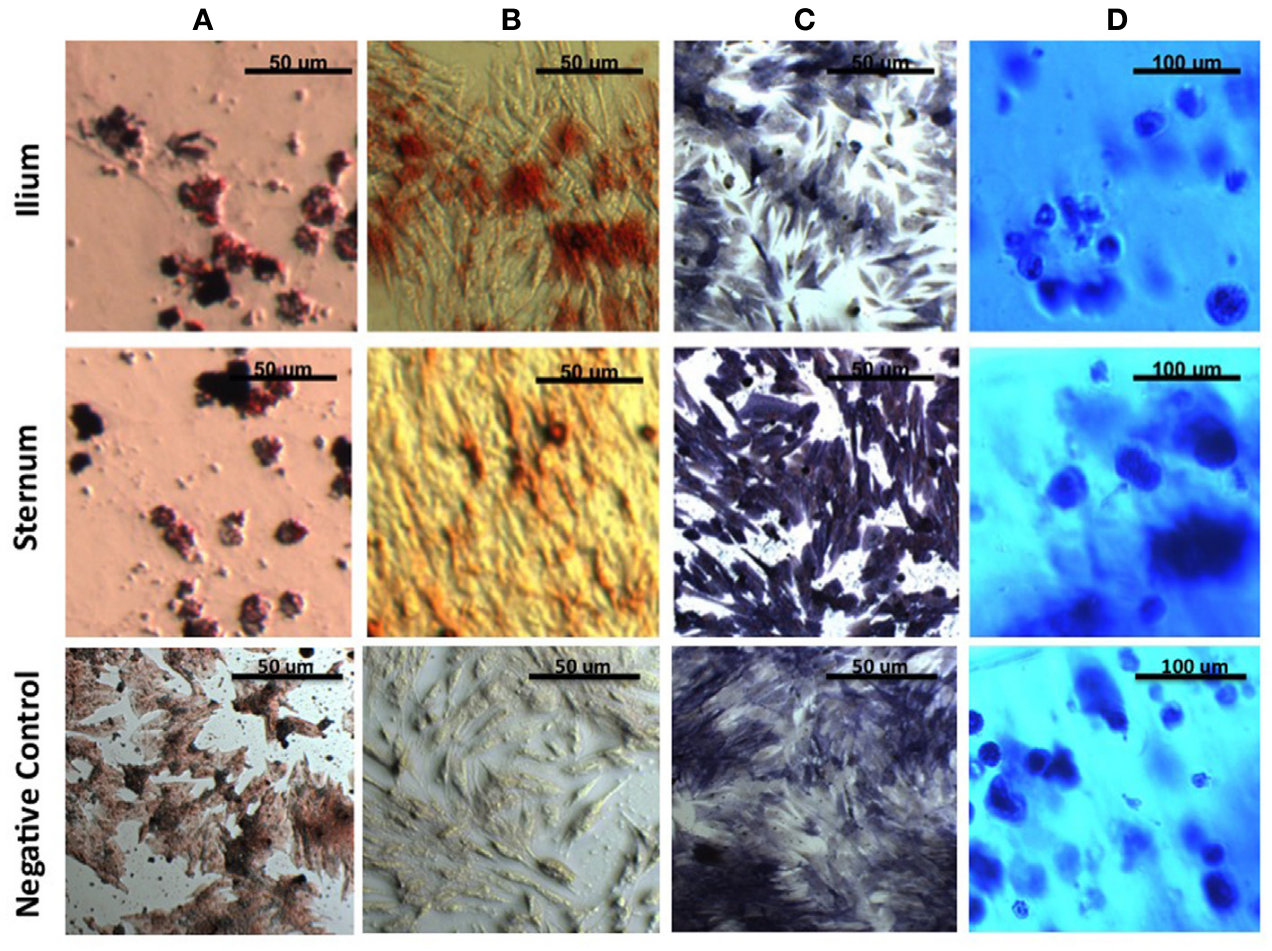
**MSCs in culture stained to confirm morphologic change**. **(A)** Oil Red O to detect lipid deposits in adipogenic culture. **(B)** Alizarin Red to detect mineralization and **(C)** Alkaline Phosphatase to detect enzymatic activity in osteogenic culture. **(D)** Toluidine blue to detect extracellular matrix in agarose chondrogenic culture. All four stains confirm morphologic change of MSCs *in vitro*.

After induction of all cell lineages, morphological changes were noted in cells from both equine sternum and ilium (Figure 1). Adipogenic cells became less fibroblastic and Oil Red O staining resulted in dark red lipid staining in which the treated cells produced higher percentages of fatty aggregates, and the negative controls maintained their fibroblastic appearance. Osteogenic cells became cuboidal in shape, and nodules became apparent. Alkaline phosphatase and Alizarin Red stains both highlighted morphological changes associated with osteogenic differentiation. Chondrogenic cultures became less friable than the negative controls when sliced for staining. Toluidine blue staining allowed us to confirm chondrogenic changes as GAG would stain blue when viewed under the microscope. No subjective differences between iliac and sternal samples were observed.

**Figure 2:**
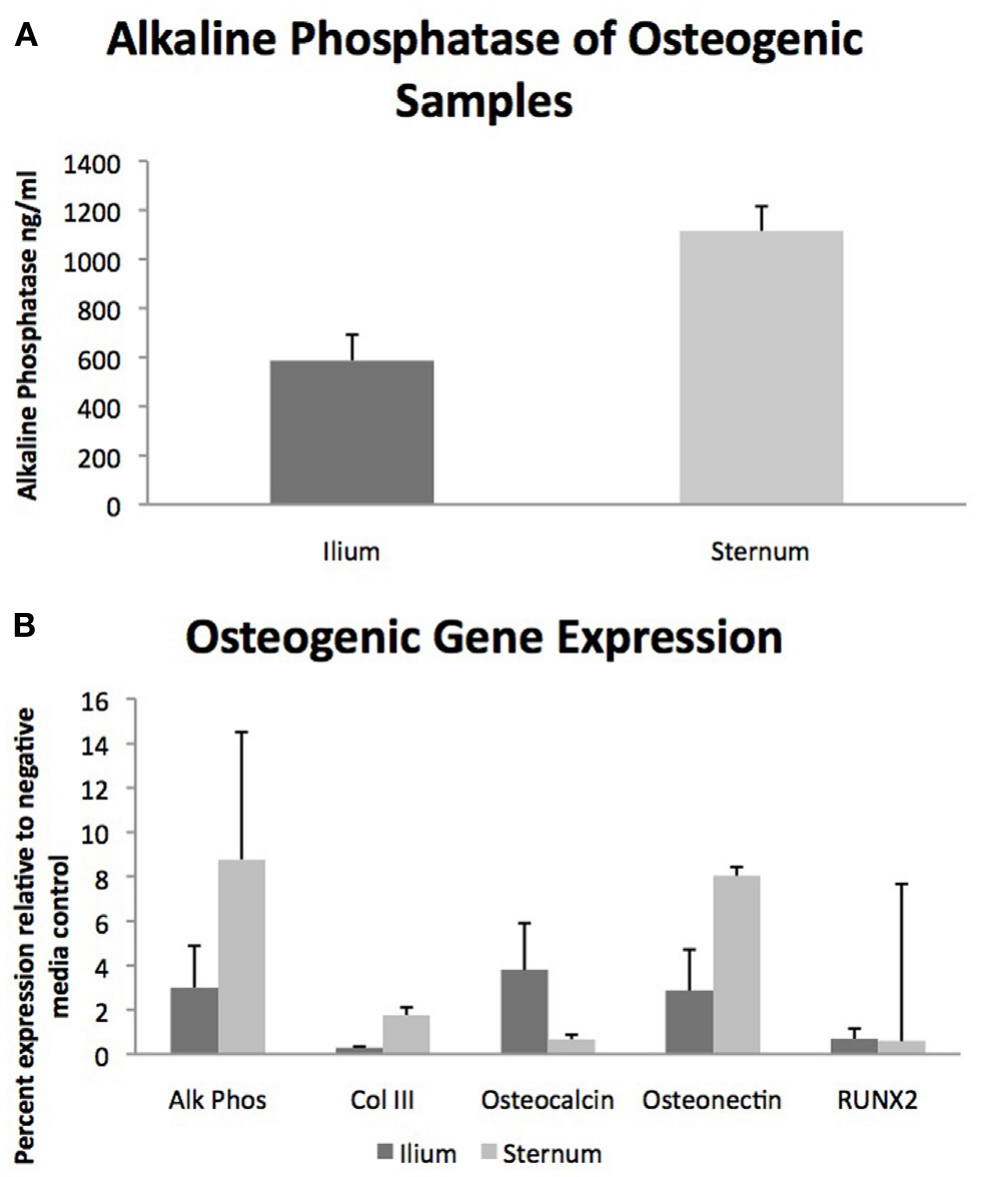
**Osteogenic differentiation of MSCs from sternum and ilium (*n* = 9)**. **(A)** Quantifiable alkaline phosphatase produced *in vitro* culture was not found to be significantly different between sternum (1115.9 ng/ml ± ST error 100.38) and ilium samples (586.5 ng/ml ± ST error 105.8). **(B)** Expression of genes associated with osteogenic change were not found to be statistically significant across treatment groups. Alk Phos expression of ilium (3.01 ± ST error 1.87) and sternum (8.77 ± ST error 1.08). Col III expression of ilium (0.30 ± ST error 0.04) and sternum (1.77 ± ST error 0.39). Osteocalcin expression of ilium (3.75 ± ST error 2.10) and sternum (0.67 ± ST error 0.205). Osteonectin expression of ilium (2.87 ± ST error 1.84) and sternum (8.04 ± ST error 5.73). RUNX2 expression of ilium (0.70 ± ST error 0.45) and sternum (0.59 ± ST error 0.34).

Quantifiable osteogenic characteristics of cells were not found to be different (Figure 2) including alkaline phosphatase levels (*p* = 0.31) and the gene expression of Alk Phos (*p* = 0.46), Col III (*p* = 0.27), Osteocalcin (*p* = 0.21), Osteonectin (*p* = 0.44), and RUNX2 (*p* = 0.86).

**Figure 3:**
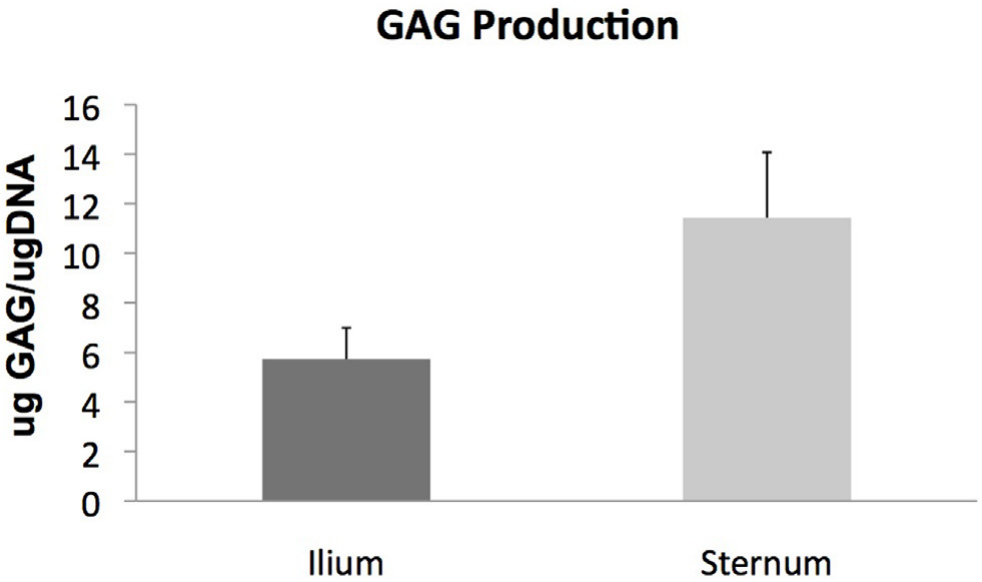
**Chondrogenic differentiation of MSCs from sternum and ilium (*n* = 9)**. Quantifiable GAG produced in *in vitro* culture normalized to DNA of cells was not found to be statistically different between ilium (5.721 μg GAG/μgDNA ± ST error 1.26) and sternum (11.43 μg GAG/μgDNA ± ST error 2.64).

**Figure 4:**
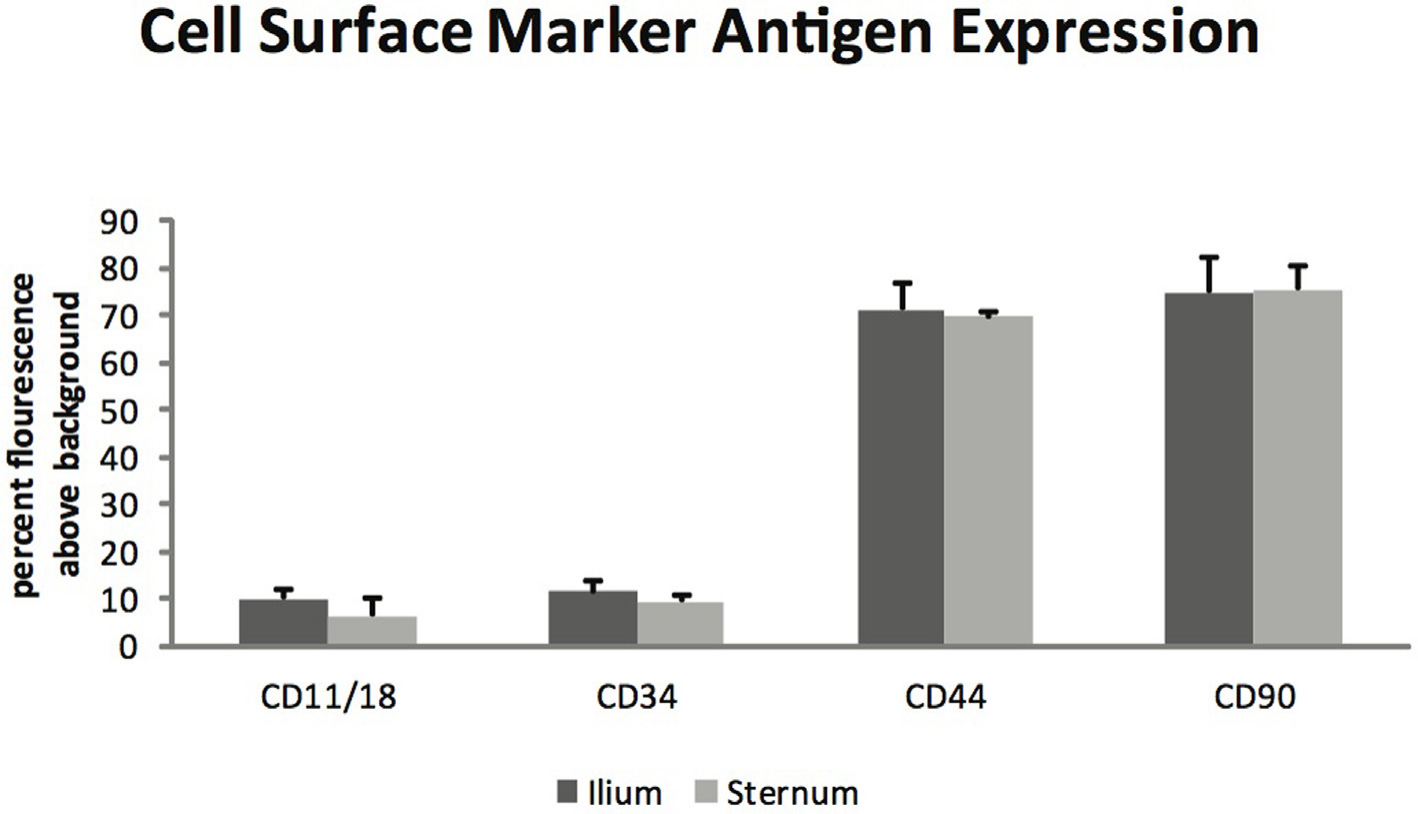
**Cell surface marker antigen expression of passage three MSCs from ilium and sternum (*n* = 9)**. Cell surface marker antigen expression was not found to be different across aspiration locations. Values in percent flourescence above background for CD11a/18 ilium (9.87 ± ST error 1.73) and sternum (6.34 ± ST error 1.08), CD34 ilium (11.29 ± ST error 2.36) and sternum (9.19 ± ST error 1.63), CD44 ilium (71.20 ± ST error 5.50) and sternum (69.46 ± ST error 3.70), CD90 ilium (74.70 ± ST error 7.16) and sternum (75.34 ± ST error 4.92).

Glycosaminoglycan content in chondrogenic cultures was normalized to DNA and was not found to be different between aspiration locations (*p* = 0.14) (Figure 3). Cell surface marker antigen expression of CD11a/18 (*p* = 0.07), CD34 (*p* = 0.34), CD44 (*p* = 0.72) and CD90 (*p* = 0.95) was not significantly different between iliac and sternal cells (Figure 4).

**Figure 5:**
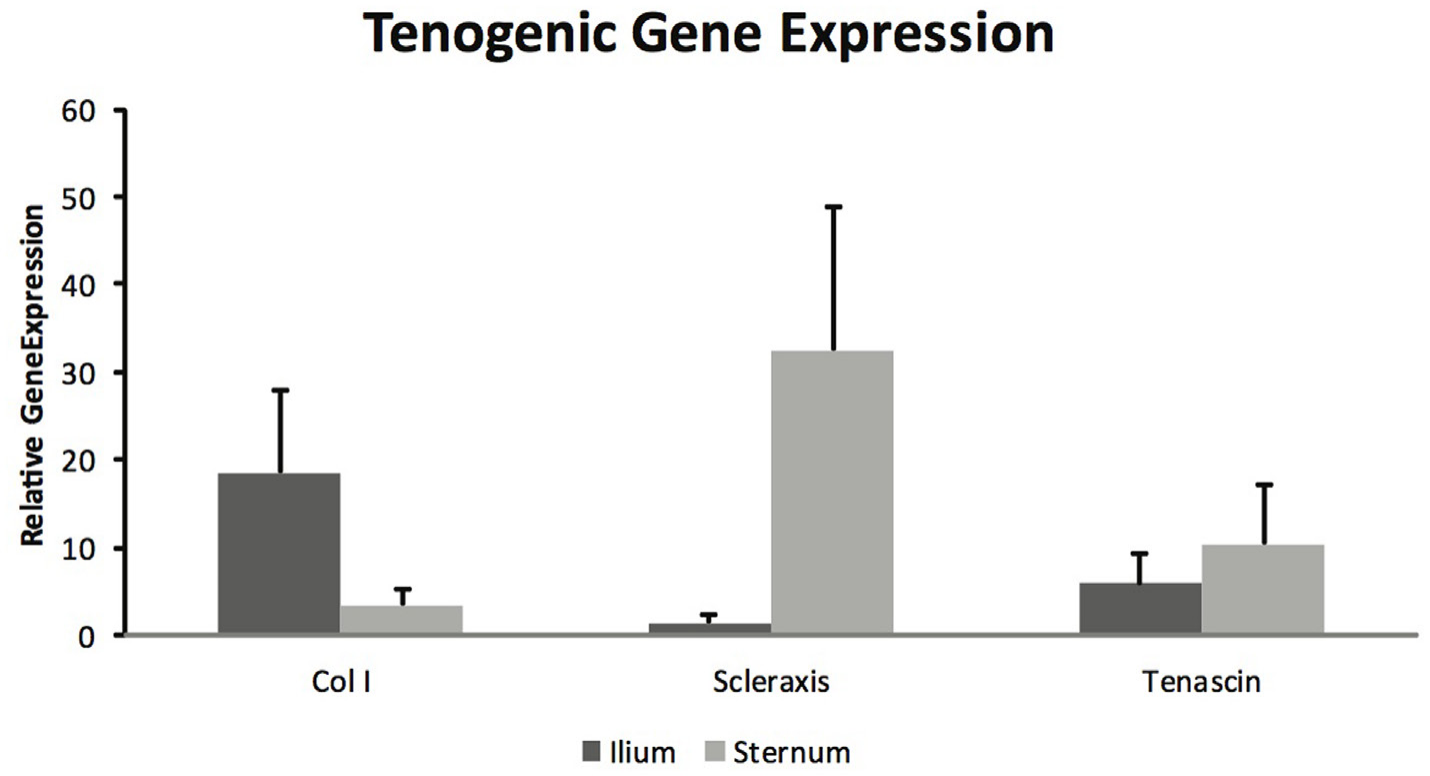
**Tenogenic gene expression of samples from ilium and sternum were not found to be statistically significantly different at 21 days of cell culture (*n* = 9)**. Relative gene expression: collagen ilium (18.51 ± ST error 9.37) and sternum (3.42 ± ST error 1.93), scleraxis ilium (1.445 ± ST error 1.04) and sternum (35.26 ± 16.24), tenascin ilium (5.84 ± ST error 14.38) and sternum (10.46 ± ST error 26.64).

Mesenchymal stem cells from sternum and ilium cultured to become tenogenic were not found to be different in gene expression for Col I (*p* = 0.18), scleraxis (*p* = 0.08), and tenascin (*p* = 0.59) (Figure 5). The differences between sternum and ilium in scleraxis expression were not significant (*p* = 0.08).

**Figure 6:**
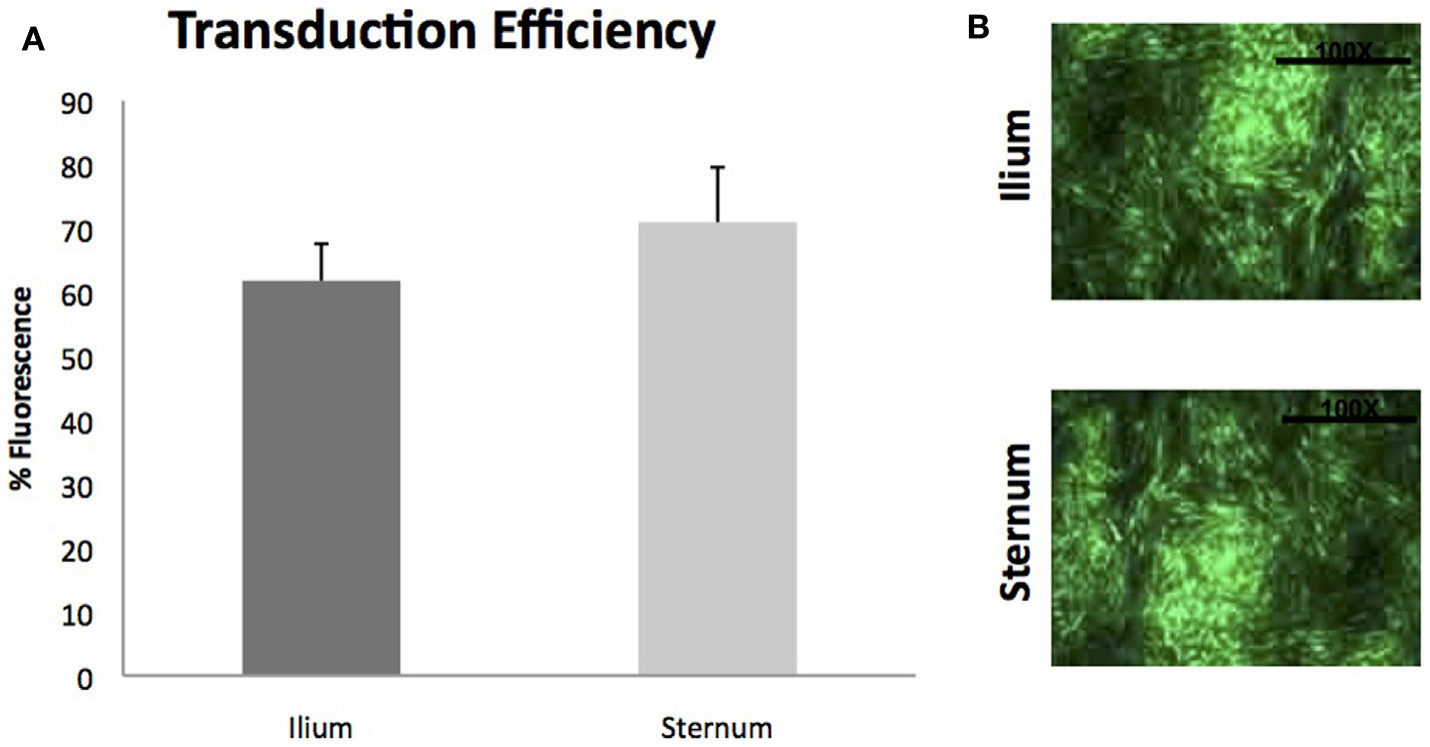
**Viral transduction of BMDMSCs with scAAV2GFP (*n* = 9)**. **(A)** Viral transduction efficiencies of MSCs cultured from ilium (61.81% fluorescence ± ST error 5.78) and sternum (70.97%fluorescence ± ST error 8.59) were not found to be different when transduced with scAAV2GFP. **(B)** Cells from sternum and ilium after gene transduction with scAAV2GFP.

Gene transduction efficiencies of MSCs between sternum and ilium using scAAV2GFP were also not found to be statistically significantly different (*p* = 0.42) (Figure 6). Cell morphology was similar post-gene modification and fluorescence as measured by flow cytometry was not different between sites.

## Discussion

Equine MSCs *in vitro* are well known to expand and differentiate into multiple cell lineages. The objective of the present study was to compare cell characteristics of MSCs acquired from bone marrow aspirates from equine sternum and ilium.

First, we observed that both cell populations adhere to plastic tissue culture surfaces and will expand. Previous literature is inconsistent with regard to growth rates of BMDMSCs derived from sternum and ilium, with reports indicating that there are no differences (8), that ilium proliferate faster in younger horses (9) and that sternal cells proliferate significantly faster *in vitro* than ilial cells in middle-aged horses (10). Our study began with subjective differences in multi-lineage cellular morphology. There were no observed differences in BMDMSCs treated from sternum and ilium. Thus, the ability of these cells to become adipogenic, chondrogenic and osteogenic are consistent with prior studies examining differentiation capacities *in vitro* for BMDMSCs acquired from either the sternum or from ilium (4, 22, 23).

With respect to osteogenic differentiation, quantification was not found to be significantly different across aspiration locations when evaluating total alkaline phosphatase production and relative gene expression of osteogenic genes Alk Phos, Col III, Osteocalcin, Osteonectin, and RUNX2 (Figure 2). Multiple studies have examined BMDMSC propensity to become bone *in vitro* (22, 23) but few have quantified differences in cells cultured from sternum and ilium (9). Our osteogenic quantification assays confirm the findings of Kisiday et al. in that both aspiration locations produce MSCs capable of similar osteogenic potential (9).

Chondrogenic potential of MSCs cultured from sternum and ilium was quantified using total GAG production normalized to DNA. Our results indicate no statistically significant difference in cells cultured from 5 ml marrow aspirates from equine sternum and ilium (Figure 3). We also found no difference in aggrecan and Col II expression in MSCs cultured from sternum and ilium (data not shown). Prior studies have used Col II as a reliable marker for chondrogenic differentiation *in vitro* (24), therefore our results confirm chondrogenic change occurred and was no difference in cells acquired from sternum and ilium. The similar chondrogenic propensity of sternal or ilial MSCs in this study differ from Kisiday et al. in which aspirates from the ilium were found to produce 29% more GAG *in vitro* when MSCs were placed in agarose (9). In that study, both 5 and 50 ml aspirates were compared between sternum and ilium and while no differences existed in small and large volume draws location effect of ilium influenced measured GAG levels.

Flow cytometry analysis revealed that MSCs from both sternum and ilium aspirates expressed CD90 and CD44 and lacked expression of CD11a/CD18 and CD34 (Figure 4). CD44 is an equine specific antibody and has been associated with a variety of cell types including lymphocytes, monocytes, granulocytes, erythrocytes, and fibroblasts (12, 25). CD90 is produced against canine specifically but cross reacts to equine antibodies and is also noted for its hematopoietic stem cell subsets including neurons, fibroblasts, and stromal cells (12, 26). CD11a/CD18 in human medicine has been associated with monocyte migration and adhesion of leukocytes (27, 28). Finally, CD34 has been associated with neurons and related lesions in humans (12, 29, 30) and hematopoiesis and platelet formation in human ­progenitor cells (30).

There have been multiple markers investigated for stemness (11–13) as there is not a specific monoclonal antibody currently used to determine multi-lineage potential in the equine as there have been in humans. One of the major difficulties in equine MSC research is the low number of specific monoclonal antibodies available due to the fact that the human specific monoclonal antibodies do not cross react with the equine species (12, 31, 32). As bone marrow aspirate mononuclear cells become established rather than freshly isolated, they tend to have a more uniform positive expression of CD44 and CD29 (12, 31). Furthermore, established MSC cultures tended to increase in CD90 expression of intensity of fluorescence (12, 31). MSCs in culture have also been found to have decreased expression of CD11a/CD18 and CD45RB over time (12). The general consensus is that there is not a single monoclonal antibody currently that indicates equine MSC quality, but rather a collection that help to establish multipotency (12, 31, 32).

Our results are consistent with previous studies, suggesting that as MSC cultures become more homogeneous the population of cells decreases expression of CD11/18, CD34, and CD45 while increasing expression of CD44, CD90, CD117, and CD13 (12, 13, 31). The present study focused on positive expression of CD44 and CD90 with decreased expression of CD11/18 and CD34 and found no statistically significant difference in MSCs acquired from equine sternum or ilium.

A previous study also comparing 5 ml samples from ilium and sternum revealed no statistical differences in nucleated cell counts and growth rates in young horses and our data suggest that the cells do not differ in their quality or trilineage potential (8). As referenced previously, a study examining large and small volume aspirates (50 and 5 ml) did find that ilial aspirates had higher proliferation rates *in vitro* and greater chondrogenic propensity (9). Another recent study investigating cell growth rates found that there were significantly higher growth rates in aspirates from the sternum in middle-aged horses (10). This study concluded that there are less viable cells aspirated from equine ilium in middle-aged horses. Perhaps cells from the sternum and ilium change their growth and multi-lineage propensity over the age of the horse, and this change is worthy of further investigation.

Equine MSCs have been used clinically to treat a number of tendon injuries, and have been successful (2, 15, 16). Several studies have demonstrated MSCs capacity to become tenogenic *in vitro* (6, 19). A major aim of this project was to determine if there were differences in BMDMSC’s propensity to become tenogenic when acquired from sternum and ilium. BMDMSCs cultured in tenogenic media were lifted after 21 days and gene expression was quantified for Col I, Scleraxis and Tenascin (Figure 5) (19). There were no statistically significant differences between sternum and ilium; however, the differences in scleraxis expression between sternum and ilium was approaching a significant value (*p* = 0.08). Scleraxis is important as it is a specific marker for tendons and ligaments (33). Previous studies have indicated that scleraxis is essential in tendon differentiation and force transmission (34). Our data illustrate a trend of cells from the sternum to express higher levels of scleraxis, potentially indicative of tenogenic activity.

Finally, utilizing growth factors to enhance healing has been shown to enhance repair of musculoskeletal tissues (21, 35). Viral gene therapy vectors have been shown to result in efficient enhancement of growth factors in the sites of orthopedic injuries, and MSCs offer a unique target. Our data examined BMDMSCs from sternum and ilium and their viral transduction efficiencies quantified by fluorescence of a green protein (GFP). We did not find a statistically significant difference in gene transduction potential between BMDMSCs derived from sternum or ilium. Studies examining equine BMDMSC transduction have used sternal or ilium aspirates exclusively, both with transduction success (23, 35). Our data are the first to suggest that there is no difference between aspiration sites in gene transduction efficiencies.

The hypothesis that there would be no significant difference of BMDMSCs harvested from sternal and ilial aspiration sites with regard to trilineage potential, tenogenesis, cell surface markers, and gene therapeutic potential was proven, although a trend for increased scleraxis existed for sternal samples. Future studies examining cell characteristics when MSCs are placed *in vivo* will determine if *in vitro* results closely correlate with *in situ* characteristics.

## Conclusion

This study suggests that equine MSCs derived from the sternum and the ilium have robust chondrogenic, osteogenic, and tenogenic capacity. Future studies are needed on the differences in levels of scleraxis noted. The cell surface markers are not significantly different between aspiration locations, and gene transduction efficiencies reveal that they are also good candidates for gene therapeutic applications. Further *in vivo* studies are needed to determine actual clinical significance of these data.

## Conflict of Interest Statement

The authors have declared the following competing interests: Drs. Laurie R. Goodrich, John David Kisiday, and C. Wayne McIlwraith have financially invested in Advanced Regenerative Therapies, Inc. The remaining authors declare that the research was conducted in the absence of any commercial or financial relationships that could be construed as a potential conflict of interest.

## References

[1] Rossdale PD Hopes R Digby NJ. Epidemiological study of wastage among racehorses 1982 and 1983. Vet Rec (1985) 116:66–9. 10.1136/vr.116.3.66 3976145

[2] Smith RKW Korda M Blunn GW Goodship AE Isolation and implantation of autologous equine mesenchymal stem cells from bone marrow into superficial digital flexor tendon as a potential novel treatment. Equine Vet J (2003) 35:99–102. 10.2746/042516403775467388 12553472

[3] Frisbie DD Kisiday JD Kawcak CE Werpy NM McIlwraith CW. Evaluation of adipose-derived stromal vascular fraction or bone marrow-derived mesenchymal stem cells for treatment of osteoarthritis. J Orthop Res (2009) 27:1675–80. 10.1002/jor.20933 19544397

[4] Vidal MA Kilroy GE Johnson JR Lopez MJ Moore RM Gimble JM Cell growth characteristics and differentiation frequency of adherant equine bone marrow-derived mesenchymal stem cells: adipogenic and ostogenic capacity. Vet Surg (2003) 35:601–10. 10.1111/j.1532-950X.2006.00197.x 17026544

[5] Vidal MA Robinson SO Lopez SO Paulsen DB Borkhsenious O Johnson JR Comparison of chondrogenic potential in equine mesenchymal stromal cells derived from adipose tissue and bone marrow. Vet Surg (2008) 37:713–24. 10.1111/j.1532-950X.2008.00462.x 19121166 PMC2746327

[6] Burk J Ribitsch I Gittel C Juelke H Kasper C Staszyk C Growth and differentiation characteristics of equine mesenchymal stromal cells derived from different sources. Vet J (2013) 195:98–106. 10.1016/j.tvjl.2012.06.004 22841420

[7] Kisiday JD Kopesky PW Evans CH Grodzinsky AJ McIlwraith CW Frisbie DD Evaluation of adult equine bone marrow and adipose-derived progenitor cell chondrogenesis in hydrogel cultures. J Orthop Res (2008) 26:322–31. 10.1002/jor.20508 17960654

[8] Adams MK Goodrich LR Rao S Olea-Popelka F Phillips N Kisiday JD Equine bone marrow-derived mesenchymal stem cells (MSCs) from the ilium and sternum: are there differences? Equine Vet J (2012) 45(3):372–5. 10.1111/j.2042-3306.2012.00646.x 23009322 PMC3581704

[9] Kisiday JD Goodrich LR McIlwraith WC Frisbie DD Effects of equine bone marrow aspirate volume on the isolation, proliferation, and differentiation potential of mesenchymal stem cells. Am J Vet Res (2013) 74:801–7. 10.2460/ajvr.74.5.801 23627395

[10] Delling U Lindner K Ribitsch I Jülke H Brehm W. Comparison of bone marrow aspiration at the sternum and the tuber coxae in middle-aged horses. Can J Vet Res (2012) 76:52–6. 22754095 PMC3244288

[11] De Schauwer C Meyer E Van de Walle GR Van Soom A. Markers of stemness in equine mesenchymal stem cells: a plea for uniformity. Theriogenology (2011) 75:1431–43. 10.1016/j.theriogenology.2010.11.008 21196039

[12] Radcliffe CH Flaminio MJBF Fortier LA. Temporal analysis of equine bone marrow aspirate during establishment of putative mesenchymal progenitor cell populations. Stem Cells Dev (2010) 19(2):269–81. 10.1089/scd.2009.0091 19604071 PMC3138180

[13] Martinello T Bronzini I Maccatrozzo L Iacopetti I Sampaolesi M Mascarello F Cryopreservation does not affect the stem characteristics of multipotent cells isolated from equine peripheral blood. Tissue Eng (2010) 16:771–81. 10.1089/ten.TEC.2009.0512 19839741

[14] Kryger GS Alphonsus KS Chong MD Costa M Pham H Bates SJ A comparison of tenocytes and mesenchymal stem cells for use in flexor tendon tissue engineering. J Hand Surg Am (2007) 32A:597–605. 10.1016/j.jhsa.2007.02.018 17481995

[15] Smith RKW. Mesenchymal stem cell therapy for equine tendinopathy. Disabil Rehabil (2008) 30:1752–8. 10.1080/09638280701788241 18608378

[16] Godwin EE Young NJ Dudhia J Beamish IC Smith RKW Implantation of bone marrow-derived mesenchymal stem cells demonstrates improved outsome in horses with overstrain injury of the superficial digital flexor tendon. Equine Vet J (2012) 44:25–32. 10.1111/j.2042-3306.2011.00363.x 21615465

[17] Hoffman A Gross G. Tendon and ligament engineering in the adult organism: mesenchymal stem cells and gene-therapeutic approaches. Int Orthop (2007) 31:791–7. 10.1007/s00264-007-0395-9 17634943 PMC2266662

[18] Butler DL Juncosa-Melvin GP Galloway MT Shearn JT Gooch C Awad H. Functional tissue engineering for tendon repair: a multidisciplinary strategy using mesenchymal stem cells, bioscaffolds, and mechanical stimulation. J Orthop Res (2008) 26:1–9. 10.1002/jor.20456 17676628

[19] Violini S Ramelli P Pisani LF Gorni C Mariani P. Horse bone marrow mesenchymal stem cells express embryo stem cell markers and show the ability for tenogenic differentiation by in vitro exposure to BMP-12. BMC Cell Biol (2009) 10:29. 10.1186/1471-2121-10-29 19383177 PMC2678092

[20] Cheng X Yang T Meng W Liu H Zhang T Shi R. Overexpression of GDF5 through an adenovirus vector stimulates osteogenesis of human mesenchymal stem cells in vitro and in vivo. Cells Tissues Organs (2012) 196:56–67. 10.1159/000330791 22287558

[21] Nixon AJ Goodrich LR Scimeca MS Witte TH Schnabel LV Watts AE Gene therapy in musculoskeletal repair. Ann NY Acad Sci (2007) 1117:310–27. 10.1196/annals.1402.065 18056051

[22] Arnhold SJ Goletz I Klein H Stumpf G Beluche LA Rohde C Isolation and characterization of bone marrow-derived equine mesenchymal stem cells. AJVR (2001) 68(10):1095–105. 10.2460/ajvr.68.10.1095 17916017

[23] Carpenter RS Goodrich LR Frisbie DD Kisiday JD Carbone B McIlwraith CW Osteoblastic differentiation of human and equine bone marrow-derived mesenchymal stem cells with BMP-2, BMP-7 or BMP-2/7 genetic modification in the presence and absence of dexamethasone. J Orthop Res (2010) 2:1330–7. 10.1002/jor.21126 20309952 PMC3200399

[24] Worster AA Nixon AJ Brower-Toland BD Williams J. Effect of transforming growth factor beta1 on chondrogenic differentiation of cultured equine mesenchymal stem cells. AJVR (2000) 61:1003–10. 10.2460/ajvr.2000.61.1003 10976727

[25] Tavernor AS Deverson EV Coadwell WJ Lunn PD Zhang C Davis W Molecular cloning of equine CD44 cDNA by a COS cell expression system. Immunogenetics (1993) 37:474–7. 10.1007/BF00222474 8436424

[26] Kamishina K Deng J Takashi O Cheeseman JA Clemmons RA. Expression of neural markers on bone marrow-derived canine mesenchymal stem cells. AJVR (2006) 67:1921–8. 10.2460/ajvr.67.11.1921 17078756

[27] Shang XZ Issekutz AC. Contribution of CD11a/CD18, CD11b/CD18, ICAM-1 (CD54) and -2 (CD102) to human monocyte migration through endothelium and connective tissue fibroblast barriers. Eur J Immunol (1998) 28:1970–9. 10.1002/(SICI)1521-4141(199806)28:06<1970::AID-IMMU1970>3.0.CO;2-H 9645379

[28] Furie MB Tancinco MC Smith CW Monoclonal antibodies to leukocyte integrins CD11a/CD18 and CD11b/CD18 or intercellular adhesion molecule-1 inhibit chemoattractant-stimulated neutorphil transendothelial migration in vitro. Blood (1991) 78:2089–97. 1680499

[29] Weiss SW Nickoloff BJ. CD-34 is expressed by a distinctive cell population in peripheral nerve, nerve sheath tumors, and related lesions. Am J Surg Pathol (1993) 17:1039–45. 10.1097/00000478-199310000-00009 7690524

[30] Cheng L Qasba P Vanguri P Theide MA Human mesenchymal stem cells support megakaryocyte and pro-platelet formation from CD34+ hematopoietic progenitor cells. J Cell Physiol (2000) 184:58–69. 10.1002/(SICI)1097-4652(200007)184:1<58::AID-JCP6>3.0.CO;2-B 10825234

[31] de Mattos Carvalho M Alves ALG Golim MA Moroz A Hussni CA de Oliveira PGG Isolation and immunophenotypic characterization of mesenchymal stem cells derived from equine species adipose tissue. Vet Immunol Immunopathol (2009) 32:303–6. 10.1016/j.vetimm.2009.06.014 19647331

[32] Taylor SE Smith RKW Clegg PD. Mesenchymal stem cell therapy in equine musculoskeletal disease: scientific fact or clinical fiction? Equine Vet J (2007) 39:172–80. 10.2746/042516407X180868 17378447

[33] Schweitzer R Chyung JH Murtaugh LC Brent AE Rosen V Olson E Analysis of the tendon cell fate using scleraxis, a specific marker for tendons and ligaments. Development (2001) 128:3855–66. 11585810 10.1242/dev.128.19.3855

[34] Murchison ND Price BA Conner DA Keene DR Olson EN Tabin CJ Regulation of tendon differentiation by scleraxis distinguishes force-transmitting tendons from muscle-anchoring tendons. Development (2007) 134:2697–708. 10.1242/dev.001933 17567668

[35] Schnabel LV Lynch ME Van der Meulen MCH Yeager AE Kornatoswski MA Nixon AJ Mesenchymal stem cells and insulin-like growth factor-1 gene-enhanced mesenchymal stem cells improve structural aspects of healing in equine flexor digitorum superficialis tendons. J Orthop Res (2009) 27:1392–8. 10.1002/jor.20887 19350658

